# Global health priorities – priorities of the wealthy?

**DOI:** 10.1186/1744-8603-1-6

**Published:** 2005-04-22

**Authors:** Eeva Ollila

**Affiliations:** 1 Globalism and Social Policy Programme (GASPP), Welfare Research Group, National Research and Development Centre for Welfare and Health (Stakes), Helsinki, Finland

## Abstract

Health has gained importance on the global agenda. It has become recognized in forums where it was once not addressed. In this article three issues are considered: global health policy actors, global health priorities and the means of addressing the identified health priorities. I argue that the arenas for global health policy-making have shifted from the public spheres towards arenas that include the transnational for-profit sector. Global health policy has become increasingly fragmented and verticalized. Infectious diseases have gained ground as global health priorities, while non-communicable diseases and the broader issues of health systems development have been neglected. Approaches to tackling the health problems are increasingly influenced by trade and industrial interests with the emphasis on technological solutions.

## Global health policy actors

The major actors in global health policy are changing. New actors are entering and old ones are losing power; the overall change has seen a shift from global nation-based health-policy-making structures towards more diversity that puts emphasis on private sector actors. In the 1980s and 1990s there was a shift in global health policy making from the UN agencies towards financial institutions. This shift has meant increasing attention being given to involving private actors in health policy [1-4]. Towards the end of the 20^th ^century the UN increasingly collaborated with business, which subsequently increased the influence of private interests in the UN system. [5-8]. This development was partly due to the declining levels of development assistance of the OECD (Organisation for Economic Co-operation and Development) countries to the UN, which became particularly acute in the 1990s [9], and partly due to the fear that the UN would become marginalized if it did not increase its collaboration with the corporate sector, which had gained power in overall policy-making [10].

In the UN forums, civil society has become recognized as an important body of actors in global policy-making, as seen at the UN Conference for Environment and Development in 1992, and at the International Conference on Population and Development in 1994, where women's organisations were instrumental in shaping the Programme of Action. Regarding health matters, the not-for-profit sectors of the civil society have played an important role for much longer, most notably in the debates concerning essential drugs, breast milk substitutes, and weaning foods in the 1970s and 1980s. [11]. More recently the public health NGOs have been important, for example, in shaping pharmaceutical policies and emphasising the needs and rights of HIV-infected people.

The emergence of new global health policy actors – as a result of new global legally independent public-private entities such as the Global Alliance for Vaccines and Immunizations (GAVI), the Global Fund to Fight AIDS, Malaria and Tuberculosis (GFATM) and the Global Alliance for Improved Nutrition (GAIN) – to address selected health issues at the turn of the century has further diversified the global health policy scene. Furthermore, new challenges in health research have been defined under the public-private partnership umbrella of the Global Forum for Health Research.

Development aid to health has continued to grow substantially since 1992 despite the fall in total official development assistance (ODA) since that time. The USA provides about one third of the total bilateral aid to health. Other bilateral donors are substantially smaller. The multilateral agencies provide one third of the total official development assistance to health and of that assistance 80% comes from the International Development Association (IDA) [12]. As a new funding source, the Global Health programme of the Bill and Melinda Gates Foundation (BMGF) has become not only significant in size, but also in setting health policy. The funding from the USA, IDA and the BMGF are of about the same order.

The US role in global health policy setting has increased in the 1990s. [13] Traditionally the US AID emphases have been on fostering goals such as privatization and economic liberation, and on ties to US exports and technical assistance [14]. During the past decade, the USA has been active in lifting global health issues in new forums, such as the G8. The USA was also instrumental in the creation of the GFATM, towards which the EU, for instance, was initially more critical. According to Kagan [15], the US foreign policy is less inclined to act through international institutions such as the UN and less inclined to work co-operatively with other nations to pursue common goals, while the European foreign policy emphasis is on multilateralism over unilateralism.

## Global health priorities

Global health priorities have in recent years been defined through several processes and by several actors and at various forums. In 2000 and 2001, HIV/AIDS, tuberculosis and malaria came to be discussed in a variety of forums at the UN as well as outside the UN, and commitments to address the three diseases were made, for example, by the G8, the World Bank, the World Economic Forum and the European Commission [16,17].

Millennium Development Goals (MDGs) [18] are a product of consultations between international agencies, but were also adopted by the United Nations (UN) General Assembly in September 2001 as part of the road map for implementing the substantially broader Millennium Declaration, which it had adopted in September 2000 [19]. The MDGs have eight goals, three of which are health-focussed, namely those on child mortality, maternal health, and HIV/AIDS, malaria and other diseases.

The UN-led Millennium Project, directed by the economist Jeffrey Sachs, has the objective of ensuring that all developing countries meet the MDGs. The whole UN system has since been requested to adapt to addressing the MDGs, and to report to the Secretary General on their achievements in that direction. For health policies, this has meant, for example, pressures from some of the member states, such as the UK, for the WHO to refocus its work on the MDGs, most notably to the goal concerning HIV/AIDS, malaria and tuberculosis, while its wider mandate as the normative health organisation that sets norms and standards and promotes the building up a wider health systems would not be so emphasised [20]. The MDGs have become an important tool to steer both the UN system towards a narrower agenda with more emphasis on selected interventions and country presences, but more recently increased attention has been placed on the need for addressing development – including health policy issues and systems – more comprehensively [21-23].

Largely the same priorities for health emerged from the report of the Commission of Macroeconomics and Health (CMH) in December 2001 [24], which concluded that public health resources should be directed to the following priorities: communicable diseases; malnutrition, which exacerbates childhood infections; and maternal and perinatal mortality.

Development aid for health is also largely steered towards tackling communicable infectious [25]. USAID has financed population programmes, including family planning, for three decades, while its emphasis on health issues is more recent. In 2002, the USAID population, health, and nutrition funding covered HIV/AIDS, family planning/reproductive health, child survival/maternal health, and infectious diseases [26]. The BMGF has provided strategic funding for the founding of new structures for global health policy making – such as GAVI and GAIN – and for the implementation of the recommendations derived from the CMH. Its Global Health programme focuses on infectious disease prevention, vaccine research and development, and reproductive and child health, with emphasis on the development and implementation of technologies, though recurrent costs or chronic conditions are not financed [28]. In GAVI, the substantial BMGF funding is targeted at new vaccines. Efforts have also been made to tackle health challenges through new health technology research and development funding under the Bill and Melinda Gates Foundation funded Grand Challenges in Global Health initiative [29].

According to global mortality and burden-of-disease calculations, the above-set priorities indeed represent the majority of deaths and ill-health in sub-Saharan Africa [27], but do not represent the majority of ill-health in any other region. They cover less that a third of the global ill-health [24,27]. Today, non-communicable diseases are a cause of the majority of ill-health in developing countries, and their importance is increasing rapidly. They affect all socioeconomic groups and in many cases the risks are biggest in the poorest sections of the populations [25].

Kickbusch [13] argues that global unilateralism has linked the global health agenda to the US national interests, as well as created a systematic effort to respond to the challenge of the present US administration to show effectiveness. As a result, the four Es – economics, effectiveness, efficiency, and evidence – are now the new battle cries for the development community. Selected interventions to eradicate infectious diseases fit well with these premises.

The lists of the current global health priorities can be seen as reflecting health-related problems in the developing countries that are perceived to threaten the vital interests of industrialised countries. Linking national interests to development aid is by no means new. In the 1970s, such concerns were central in, for example, the argumentation for population programme implementation [30,31]. Nevertheless, it is noteworthy that since the mid-1990s the arguments for a greater US engagement in global health have been expressed increasingly in terms of national interests or enlightened self-interest [13,16].

The joint strategic plan of the US Department of State and the US Agency for International Development (USAID) for the fiscal years 2004–2009 states that US foreign policy and development policy are fully aligned to advance the National Security Strategy. The strategy sets out its mission as being to create a more secure, democratic and prosperous world for the benefit of the American people and the international community. The purpose of the Strategy is to help American business succeed in foreign markets and help developing countries create conditions for investment and trade [32].

Added emphasis on the trade and industrial policies has been part of global development policies. The eighth MDG is to develop global partnerships for development, which includes developing an open trading and financial system that is rule-based and non-discriminatory in co-operation with both the pharmaceutical sector, for the purpose of providing access to affordable medicines, and in co-operation with the private sector in order to make available the benefits of new technologies. The CMH also argues for increased partnerships with business [24].

## Approaches for improved global health

Health policy-making has become increasingly fragmented and verticalized, with the increasing emphases on selected interventions, the increasing number of partnerships and especially because of the founding of new entities for various health issues. Little emphasis has been put on comprehensive infrastructure building. These trends are in contrast to the stated aims of integrating health policy making with the broader development agenda or with comprehensive health sector planning.

An emphasis on innovations and innovative approaches encourages the use of new technologies and the building of new structures. Problems of unsustainability and inequity have arisen with the high levels of funding required, an emphasis on fast results, and the construction of new structures both at global and national levels [2,33-35]. In the initial faces of GAVI serious concerns were raised that those children that had been without basic vaccine coverage before GAVI funding would remain so and also be out of the reach of the new vaccines [33,36]. The GAVI emphasis on new and more expensive vaccines have raised the costs of the immunizations programmes at country level making the future financing of the programmes highly vulnerable [37].

National priorities often differ from the global priorities, and the thinking around global public goods recognizes this as a starting point. Yamey [34] has argued that the increased emphasis on global programmes and global priority setting is problematic from the point of view of national sovereignty and empowerment. He furthermore states that partnerships rarely synchronise their activities with emerging processes within countries aimed at developing their national health systems. This observation has also been made in relation to GAVI country level action [38].

Partnerships are commonly defined as voluntary and collaborative relationships between state and non-state participants who agree to work together to achieve a common purpose or undertake a specific task, and to share risks, responsibilities, resources, competencies and benefits [39]. According to Richter [7] one of the most substantive losses resulting from the shift towards the partnership paradigm is the loss of distinction between different actors in the global health arena. UN agencies, governments, transnational corporations, their business associations and public interest NGOs are all called 'partner'. The realisation that these actors have different and possibly conflicting mandates, goals and roles has been lost.

The inclusion of business as an integral part of public policy making may weaken the vital role of the public sector in norm- and standard setting and monitoring, as the public sector has been made an equal partner with business, sharing a common purpose and tasks. The WHO collaboration with business has caused harm to the credibility of the WHO's normative functions [7,40-43]. The legally independent global PPPs are structured so that public bodies with normative functions hold seats in the policy-making bodies together with business representatives both at global and national levels. This 'forced marriage' within the legally independent PPPs may harm not only the credibility of the normative functions of the regulators, but also the normative functions as such. In GAIN and in the UNFPA private sector initiative, the normative bodies are directly requested for 'supportive environments' as regards regulation, taxes and tariffs [6].

GAVI, GFATM and GAIN deal with essential health issues. Selected UN agencies (in the case of GAIN only one UN or other multilateral agency) that have mandates to deal with these health matters are invited to join their boards either as voting (GAVI and GAIN) or non-voting (GFATM) members, while industry and other private sector actors are included as full members at all levels of their structures [2,6]. The marginalisation of the UN in the structures of the legally independent global PPPs did not happen accidentally. The cautious approach of the WHO to integrating private industry into its activities has been reported as one of the main reasons for GAVI's construction as an independent legal body. Problems were encountered, for example, when issues of intellectual property rights and profits arose [44]. According to Phillips [45], the USA opposed the running of GFATM by either the UN or the World Bank. The US also demanded that the fund set up a world-wide aid-delivery system instead of relying on established agencies, such as the UN and the World Bank.

According to Stansfield et al. [46] many public sector leaders have raised the concern that in its eagerness to address market failures and pursue international public goods, PPPs are often structured so that the public sector absorbs the lion's share of the risks and costs, while the private sector absorbs a disproportionate share of the profit. On a more general note, a report by the International Monetary Fund has raised concerns over the inadequate risk-sharing in public-private partnerships [47]. This tendency can be demonstrated, for example, by the UNFPA private-sector initiative, which aimed at increasing access to reproductive health commodities. According to the initiative, governments were to give preferential tax and duty conditions and ease manufacturing and import regulations, as well as undertake and support market-related research, the donors were to provide support for marketing, advertising and marketing research, while the selected transnational contraceptive producers were requested to sell their products at affordable prices, and handle distribution and implement market-building activities. The initiative also suggested that the governments and the donors could improve the policy environment for private sector investment and security, and facilitate the building of an extensive distribution system so as to reduce the costs for the private sector. Transnational contraceptive producers were instrumental in the selection of the target developing countries, many of which had significant domestic contraceptive production [48].

## Conclusion

While globalisation increases the risk that infectious diseases travel from South to North, it has also increased the risk that major risk factors for non-communicable diseases travel from North to South. Currently, global public health policies are concentrated on selected conditions around infectious diseases and on the technological solutions for them. Addressing infectious diseases in the South is important. However, other health matters are increasingly being left for private actors to deal with. Addressing the most important risk factors of non-communicable diseases, namely tobacco, alcohol and unhealthy foods, would benefit from normative actions, including restrictions on trade and marketing [25]. Simultaneously, global health policy making is increasingly aligned with industrial and trade policies, and is being done hand in hand with business, thus weakening the firewalls necessary for effective regulation and normative actions both at global and national levels.
